# Antioxidant Peptides from Collagen Hydrolysate of Redlip Croaker (*Pseudosciaena polyactis*) Scales: Preparation, Characterization, and Cytoprotective Effects on H_2_O_2_-Damaged HepG2 Cells

**DOI:** 10.3390/md18030156

**Published:** 2020-03-11

**Authors:** Wan-Yi Wang, Yu-Qin Zhao, Guo-Xu Zhao, Chang-Feng Chi, Bin Wang

**Affiliations:** 1 Zhejiang Provincial Engineering Technology Research Center of Marine Biomedical Products, School of Food and Pharmacy, Zhejiang Ocean University, Zhoushan 316022, China; xxx0001112223@163.com (W.-Y.W.); zhaoy@hotmail.com (Y.-Q.Z.); zjou181109@163.com (G.-X.Z.); 2 National and Provincial Joint Laboratory of Exploration and Utilization of Marine Aquatic Genetic Resources, National Engineering Research Center of Marine Facilities Aquaculture, School of Marine Science and Technology, Zhejiang Ocean University, Zhoushan 316022, China

**Keywords:** redlip croaker (*Pseudosciaena polyactis*), scale, collagen, peptide, antioxidant activity

## Abstract

Bioactive peptides from fish collagens with antioxidant properties have become a topic of great interest for health, food, and processing/preservation industries. To explore the high-value utilized way of scales produced during the fish processing, collagen hydrolysates of redlip croaker (*Pseudosciaena polyactis*) scales were prepared using six different proteases, and the hydrolysate (RSCH) prepared using neutrase showed the highest degree of hydrolysis (21.36 ± 1.18%) and 2,2-diphenyl-1-picrylhydrazyl (DPPH·) radical scavenging activity (30.97 ± 1.56%) among the six hydrolysates. Subsequently, six antioxidant peptides were purified from RSCH using membrane ultrafiltration and serial chromatography, and their amino acid sequences were identified as DGPEGR, GPEGPMGLE, EGPFGPEG, YGPDGPTG, GFIGPTE, and IGPLGA with molecular masses of 629.61, 885.95, 788.96, 762.75, 733.80, and 526.61 Da, respectively. Among six collagen peptides, GPEGPMGLE, EGPFGPEG, and GFIGPTE exhibited the strongest scavenging activities on DPPH· radical (EC_50_ 0.59, 0.37, and 0.45 mg/mL), hydroxyl radical (EC_50_ 0.45, 0.33, and 0.32 mg/mL), and superoxide anion radical (EC_50_ 0.62, 0.47, and 0.74 mg/mL). GPEGPMGLE, EGPFGPEG, and GFIGPTE showed high inhibiting ability on lipid peroxidation in a linoleic acid model system and protective activities on oxidation-damaged DNA. More importantly, GPEGPMGLE, EGPFGPEG, and GFIGPTE could protect HepG2 cells from H_2_O_2_-induced oxidative damage through decreasing the levels of reactive oxygen species (ROS) and MDA and activating intracellular antioxidant enzymes of superoxide dismutase (SOD), catalase (CAT), and glutathione peroxidase (GSH-Px). These results suggested that six collagen peptides (RCP1–RCP6), especially GPEGPMGLE, EGPFGPEG, and GFIGPTE, might serve as potential antioxidants applied in nutraceutical and pharmaceutical products.

## 1. Introduction

Collagen from mammal resources is widely employed in multifarious human applications, such as food, cosmetics, pharmaceutical/biomedical, and nutraceutical products [1,2,3]. However, the application of mammal collagen and its derivatives from mammalian species arouses extensive attention and anxiety of consumers due to disease transmission-connected reasons, allergy problems, and dietary restrictions [4,5]. Therefore, collagen sources alternative to mammals are constantly investigated [6].

Marine derived collagen has the most promising perspectives as valid candidates for replacing the most commonly used mammal-derived collagen due to its large quantities and safety [7,8,9]. In addition, collagens from marine resources are acceptable for Islam, and can be used with minimal restrictions in Judaism and Hinduism [4,8]. Therefore, collagen has been extensively extracted from different fish by-products, such as skins and bones of spanish mackerel (*Scomberomorous niphonius*) [4], swim bladders of yellowfin tuna (*Thunnus albacares*) [8], swim bladders and scales of miiuy croaker (*Miichthys miiuy*) [5,10], and scales of *seabass (Lates calcarifer)* [11,12] and spotted golden goatfish (*Parupeneus heptacanthus*) [13]. However, the structural and thermal stability of marine derived collagens was weaker than those of mammal collagens due to their lower imino acid (proline and hydroxyproline) contents, which is unfavorable to its application in capsule wall and tissue materials [5,14]. Nevertheless, marine derived collagens with those undesirable characters are more easily hydrolyzed by proteases compared to mammal collagens and are suitable to be prepared for bioactive peptides [15]. Therefore, bioactive peptides prepared using marine derived collagens and gelatins have attracted broad attention due to their various promising applications [16,17,18].

Collagen peptides (CPs) from marine resources showed a variety of biological activities, such as antioxidant [19], wound healing [20], anti-aging [21], inhibiting the adipogenic differentiation [22], anti-freezing [23], iron-binding [24], and angiotensin I–converting enzyme–inhibiting [25] activities. Among those bioactive CPs, antioxidant CPs caused widespread attention because they can protect the cells and organisms from oxidative stress damage through scavenging reactive oxygen species (ROS) and upregulating the levels of intracellular antioxidant enzymes [16,18]. CPs from crimson snapper (*Lutianus erythroptrus* Block) scales [26] and royal jelly [27] could significantly prolong the average life span of Drosophila treated with H_2_O_2_ by decreasing the contents of peroxide products, such as malondialdehyde (MDA) and protein carbonylation (PCO), and improving the activity of antioxidant enzymes including total superoxide dismutase (T-SOD), and catalase (CAT), as well as by up-regulating the expression of antioxidant-related genes. In addition, CPs are popular in cosmetics because they can protect the skins from the ultraviolet (UV) injury. Liu et al. reported that CPs isolated from serum metabolites exert beneficial effects on the photoaging skin structure and collagen through activating the transforming growth factor β (TGF-β)/Smad pathway to promote procollagen synthesis and suppressing activator Protein-1 (AP-1), matrix metalloproteinase-1 (MMP-1) and MMP-3 protein expression to prevent collagen degradation [28]. Chen and Hou found that skin gelatin peptides of Pacific cod (*Gadus macrocephalus*) could repair the skin damage induced by UV radiation through inhibiting the depletion of antioxidant enzyme activity and holding back the nuclear factor kappaB (NF-κB) and pro-inflammatory cytokines expression [29]. Therefore, CPs derived from marine resources exhibit significant antioxidant activity and have a huge application potential in the food, medical, and cosmetic industries [2,18].

Fish scales, approximately fifty thousand tons per year, are produced during de-scaling processing and cause an adverse influence on the environment when they are dumped in coastal areas [30]. The amount of proteins in fish scales are ranges from 41% to 84%, which are mainly composed of collagen, keratin, and mucin [31]. Effective use of fish scales can produce high added-value products to increases economical income for the marine product processing enterprises and effectively reduce environmental pollution [10]. Therefore, collagens have been isolated from scales of some fish species [10,11,12,13]. In our previous research, acid-soluble collagen (ASC-RC) has been prepared from the scales of redlip croaker (*Pseudosciaena polyactis*), and the data of the amino acid composition, SDS-PAGE pattern, and FTIR spectrum confirmed that ASC-RC was type I collagen [32]. However, there was no information available about the preparation of bioactive peptides using scales of redlip croaker. In addition, hundreds of antioxidant peptides have been isolated from different foods resource, but it is still difficult to explain the structure–activity relationship of antioxidant peptides [18]. Therefore, the aims of this work were to (*i*) prepare and characterization the antioxidant CPs from hydrolysate of ASC-RC, and (*ii*) evaluate the in vitro antioxidant activities of isolated CPs, especially their cytoprotective effects on H_2_O_2_-damaged HepG2 cells.

## 2. Results and Discussion

### 2.1. Preparation of ASC-RC Hydrolysates and Their Radical Scavenging Activities

In the experiment, ASC-RC were hydrolyzed by pepsin, neutrase, papain, trypsin, flavoenzyme, and alcalase, respectively, and the collagen hydrolysate prepared using neutrase, referred to RSCH, showed the highest degree of hydrolysis (DH, 21.36 ± 1.18%) among the six ASC-RC hydrolysates (Table 1). Similarly, 2,2-diphenyl-1-picrylhydrazyl radical (DPPH·) scavenging activity of RSCH (30.97 ± 1.56%) was significantly stronger than those of the ASC-RC hydrolysates using other five protease at the concentration of 10 mg/mL, respectively (*p* < 0.05). Zhao et al. reported that the DH and DPPH· scavenging activity of the protein hydrolysate of Spanish mackerel prepared using in vitro gastrointestinal (GI) digestion was significantly higher than those of pepsin, neutrase, papain, trypsin, and alcalase, respectively [33]. Similarly, the DH (25.11 ± 0.67%) and hydroxyl radical (HO·) scavenging activity (85.92 ± 3.84%) of the ASC hydrolysate of croceine croaker (*P. crocea*) scales using trypsin + pepsin system was higher than those of hydrolysates using trypsin and pepsin separately [30]. The DH (13.08%), lipid peroxidation inhibition ability (48.46%), and radical scavenging activity (97.21%) of Gelatin hydrolysate of Nile tilapia (*Oreochromis niloticus*) skin using ginger protease were higher than those of the gelatin hydrolysate using pepsin-pancreatin [34]. The results in the research and previous reports indicated that the specificity of the proteases is one of the main factors for influencing the properties of bioactive peptides [33,34]. Therefore, RSCH with the yield of 18.91 ± 1.62% was chosen for next step experiment.

### 2.2. Purification of Antioxidant Peptides from Collagen Hydrolysate Prepared Using Neutrase (RSCH)

#### 2.2.1. Fractionation of RSCH

**Figure 1:**
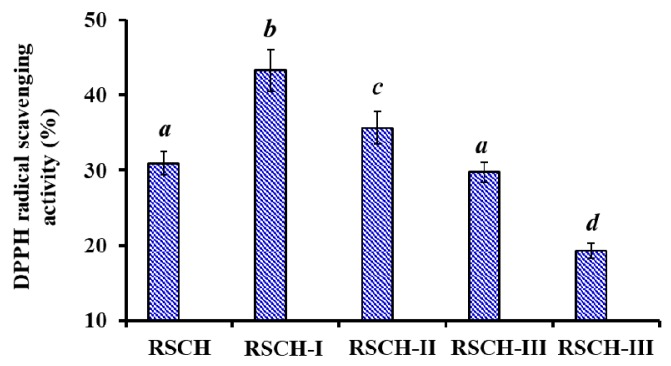
2,2-diphenyl-1-picrylhydrazyl (DPPH·) scavenging activity of RSCH and its four fractions prepared using membrane ultrafiltration at the concentration of 10.0 mg/mL. All data are presented as the mean ± SD (*n* = 3). ^a–d^ Values with the same letters indicate no significant difference (*p* > 0.05).

Using ultrafiltration with molecular weight cut off (MWCO) membranes of 1, 3 and 5 kDa, RSCH was fractionated into four fractions, including RSCH-I (<1 kDa), RSCH-II (1–3 kDa), RSCH-III (3–5 kDa) and RSCH-IV (>5 kDa). As shown in Figure 1, the DPPH· scavenging activity of RSCH-I was 43.26 ± 2.76%, which was significantly stronger than those of RSCH (30.97 ± 1.53%), RSCH -II (35.63 ± 2.18%), RSCH-III (29.74 ± 1.39%), and RSCH- IV (19.34 ± 0.98%) at the concentration of 10 mg/mL (*p* < 0.05). Peptides in collagen hydrolysates showed different antioxidant activity due to their different amino acid compositions and chain lengths [19,35]. Previous reports indicated that hydrolysate fractions with smaller MW showed stronger antioxidant activity than those of larger MW hydrolysates because peptides with a short chain length were more accessible to free radicals and easy to trap the free radical [18,35]. The present result was in line with previous reports and RSCH-I accounting for 22.54 ± 1.73% of RSCH was selected for the subsequent chromatographic separation.

#### 2.2.2. Anion-Exchange Chromatography

**Figure 2:**
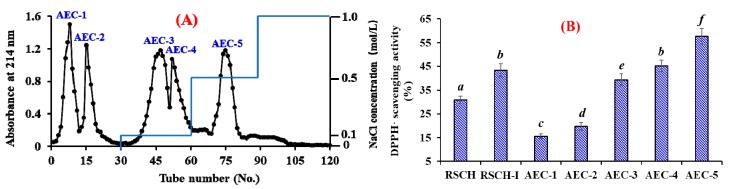
Elution profile of RSCH-I in DEAE-52 cellulose anion-exchange chromatography (**A**) and DPPH· radical scavenging activities (%) (**B**) of RSCH-I and its fractions at 10 mg/mL concentration. All data on DPPH· scavenging activity are presented as the mean ± SD (*n* = 3). (**B**) ^a–f^ Values with the same letters indicate no significant difference (*p > 0.05*).

As shown in Figure 2A, five fractions (AEC-1 to AEC-5) were separated from RSCH-I using a DEAE-52 cellulose column. Amongst them, AEC-1 and AEC-2 were eluted using deionized water (DW); AEC-3 and AEC-4 were eluted using 0.1 mol/L NaCl; and AEC-5 was eluted using 0.5 mol/L NaCl. DPPH· radical scavenging activities of RSCH-I and its five fractions are presented in Figure 2B, and the results indicated that DPPH· radical scavenging activity of AEC-5 was 57.68 ± 3.21% at the concentration of 10 mg/mL, which was significantly stronger than those of RSCH (30.97 ± 1.53%), RSCH-I (43.26 ± 2.76%), AEC-1 (15.63 ± 0.95%), AEC-2 (19.74 ± 1.57%), AEC-3 (39.34 ± 2.55%), and AEC-4 (45.16 ± 2.38%), respectively (*p* < 0.05). Bioactive peptides usually contain basic and/or acidic amino acid residues, which can be adsorbed to the anion and/or cation exchange resins, and the interaction strength depends on the number and location of the charges on the peptide sequences [18,36]. Acidic and hydrophobic amino acid residues in peptide sequences can be absorbed by the anion-exchange resins on hydrogen bonds and/or van der Waals forces. The present results indicated that AEC-5 might contain some antioxidant peptides with acidic and hydrophobic amino acid residues. Therefore, AEC-5 accounting for 10.38 ± 0.68% of RSCH-I were selected for the following experiment.

#### 2.2.3. Gel Filtration Chromatography (GFC)

**Figure 3:**
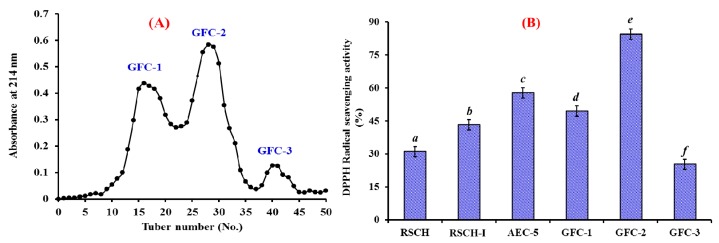
Elution profile of AEC-5 in a Sephadex G-25 column (**A**) and DPPH· radical scavenging activities (%) (**B**) of AEC-5 and its three fractions at 10 mg/mL concentration. All data on DPPH· scavenging activity are presented as the mean ± SD (*n* = 3). (**B**) ^a–f^ Values with the same letters indicate no significant difference (*p > 0.05*).

As shown in Figure 3A, AEC-5 was further fractionated into three fractions (GFC-1 to GFC-3) using a Sephadex G-25 column, and their DPPH· radical scavenging activities were presented in Figure 3B. The data indicated that DPPH· scavenging activity of GFC-2 was 84.31 ± 2.24% at the concentration of 10 mg/mL, which was significantly stronger than those of RSCH (30.97 ± 1.53%), RSCH-I (43.26 ± 2.76%), AEC-5 (57.68 ± 3.21%), GFC-1 (49.39 ± 2.35%), and GFC-3 (25.25 ± 1.02%), respectively (*p* < 0.05). GFC is a popular method for the preparation of proteins or peptides on their molecular size used in food products and pharmaceutical industries [18]. The results further proved that molecular size is one of the key factors governing the antioxidative capacity of peptides. Therefore, GFC-2 accounting for 33.45 ± 2.41% of RSCH-I was suitable for the following separation process.

#### 2.2.4. Purification of Peptides from GFC-2 by RP-HPLC

**Figure 4:**
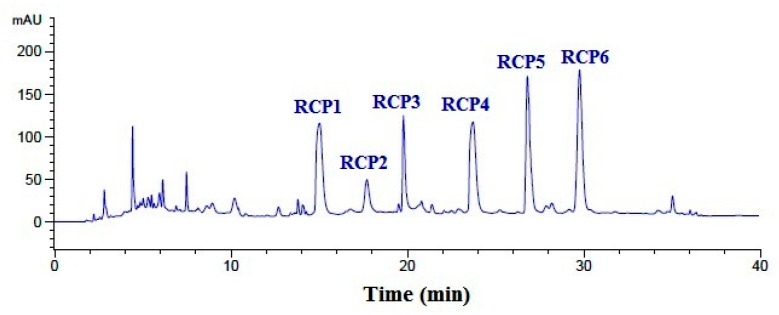
Elution profile of GFC-2 separated by RP-HPLC with an Agilent Zorbax, SB C-18 column (4.6 mm × 250 mm) from 0 to 40 min.

Finally, GFC-2 was separated by the RP-HPLC system on an Agilent 1260 HPLC system with an Agilent Zorbax C-18 column, and the eluted peptides were collected separately on the chromatographic peaks (Figure 4). On the hydrophobic property of peptides composition, six CPs with a retention time of 15.03 min (RCP1), 17.73 min (RCP2), 19.76 min (RCP3), 23.69 min (RCP4), 26.85 min (RCP5), and 29.81 min (RCP6) were prepared and lyophilized for amino acid sequence identification and activity evaluation.

### 2.3. Analysis of Amino Acid Sequences and Molecular Mass of RCP1–RCP6

As shown in Table 2, the amino acid sequences of six CPs (RCP1–RCP6) from collagen hydrolysate of redlip croaker scale were identified as Asp-Gly-Pro-Glu-Gly-Arg (DGPEGR, RCP1), Gly-Pro-Glu-Gly-Pro-Met-Gly-Leu-Glu (GPEGPMGLE, RCP2), Glu-Gly-Pro-Phe-Gly-Pro-Glu-Gly (EGPFGPEG, RCP3), Tyr-Gly-Pro-Asp-Gly-Pro-Thr-Gly (YGPDGPTG, RCP4), Gly-Phe-Ile-Gly-Pro-Thr-Glu (GFIGPTE, RCP5), and Ile-Gly-Pro-Leu-Gly-Ala (IGPLGA, RCP6), with molecular masses of 629.61, 885.95, 788.96, 762.75, 733.80, and 526.61 Da, respectively, which agreed well with their theoretical masses.

### 2.4. Antioxidant Activity

**Figure 5:**
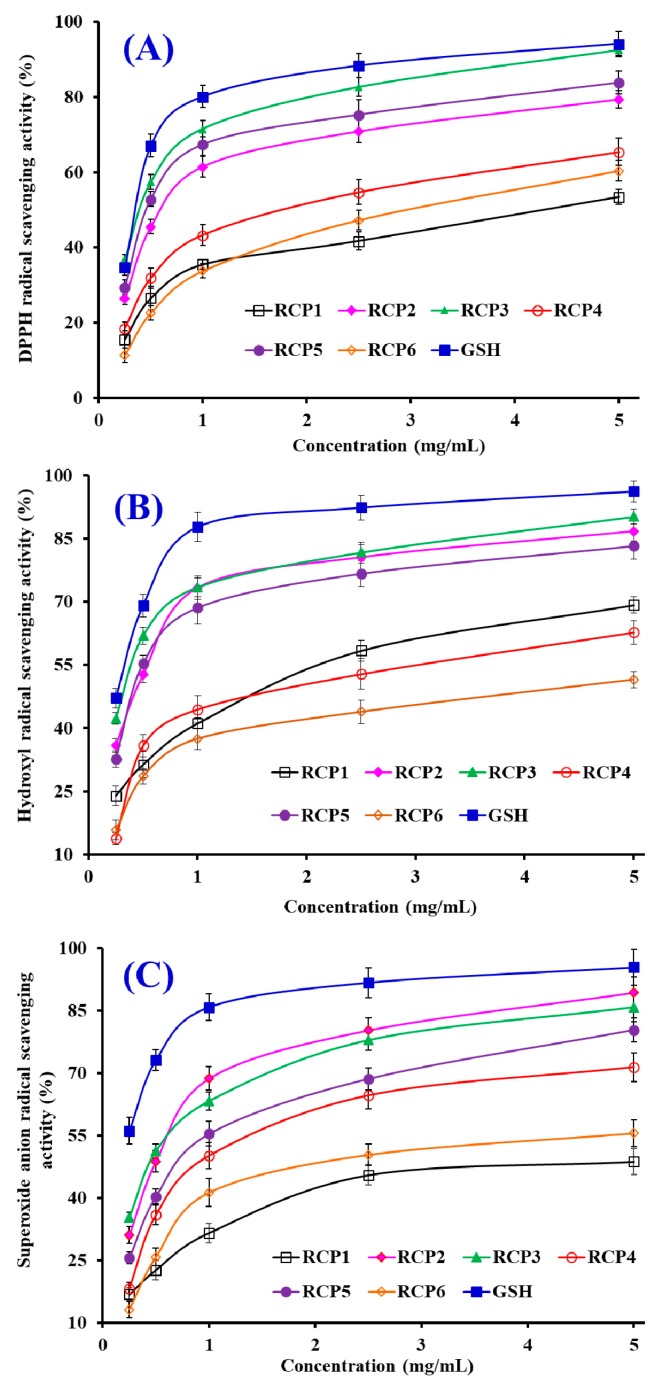
DPPH· (**A**), HO· (**B**), and $O_{2}^{-}$· (**C**) scavenging activities of six CPs (RCP1–RCP6) from collagen hydrolysate of redlip croaker (*P. polyactis*) scales. All data are presented as the mean ± SD (*n* = 3).

In the experiment, radical scavenging, lipid peroxidation inhibition, and oxidation-damaged DNA and HepG2 cells protective assays were used to evaluate the antioxidant activity of six CPs (RCP1–RCP6), and the results were presented in Table 3 and Figures 5 and 8.

#### 2.4.1. Radical Scavenging Activity

##### DPPH·Scavenging Activity

As shown in Figure 5A, six CPs (RCP1–RCP6) could strong scavenge DPPH· in a concentration-dependent manner, but their scavenging activity was less than that of the positive control of GSH at the same concentration. Table 3 indicated that the EC_50_ values of RCP2, RCP3, and RCP5 were 0.59 ± 0.06, 0.37 ± 0.05, and 0.45 ± 0.06 mg/mL, which were significantly lower than those of RCP1 (4.24 ± 0.18 mg/mL), RCP4 (1.76 ± 0.12 mg/mL), RCP6 (2.96 ± 0.17 mg/mL), respectively (*p* < 0.05). Furthermore, the EC_50_ values of RCP2, RCP3 and RCP5 were lower than those of most antioxidant peptides from protein hydrolysates of hairtail (*Trichiurus japonicas*) muscle (QNDER: 4.95 mg/mL; TKA: 1.43 mg/mL) [37], salmon pectoral fin (TTANIEDRR: 2.503 mg/mL) [38], blue mussel (*Mytilus edulis*) (YPPAK: 2.62 mg/mL) [39], blood cockle (*Tegillarca granosa*) (EPLSD: 1.23 ± 0.09 mg/mL; WLDPDG: 1.82 ± 0.16 mg/mL; EPVV: 1.13 ± 0.14 mg/mL; CYIE: 1.30 ± 0.11 mg/mL) [40], and skipjack tuna (*Katsuwonus pelamis*) scales (His-Gly-Pro-Hyp-Gly-Glu: 1.34 mg/mL) [15]. Then, six CPs (RCP1–RCP6), especially RCP2, RCP3 and RCP5, had a strong radical scavenging capability to act as a hydrogen donor for preventing the DPPH· reaction.

##### HO· Scavenging Activity

Figure 5B showed that six CPs (RCP1–RCP6) could effectively clear HO· at their concentrations between 0 and 5.0 mg/mL. The EC_50_ values of RCP2 (0.45 ± 0.05 mg/mL), RCP3 (0.33 ± 0.04 mg/mL) and RCP5 (0.42 ± 0.05 mg/mL) were significantly lower than those of RCP1 (1.65 ± 0.11 mg/mL), RCP4 (1.95 ± 0.14 mg/mL), and RCP6 (4.47 ± 0.24 mg/mL), respectively (*p* < 0.05). However, there were no significantly differences among RCP2, RCP3 and RCP5 (*p* > 0.05). Moreover, the EC_50_ values of RCP2, RCP3 and RCP5 were lower than those of antioxidant peptides from protein hydrolysates of hairtail (*T. japonicas*) muscle (His-Gly-Pro-Hyp-Gly-Glu: 1.03 mg/mL; MLGPFGPS: 0.74 mg/mL) [37], loach (*Misgurnus anguillicaudatus*) (PSYV: 2.64 mg/mL) [41], blood cockle (*T. granosa*) (EPLSD: 2.18 ± 0.16 mg/mL; WLDPDG: 1.54 ± 0.11 mg/mL; EPVV: 1.09 ± 0.08 mg/mL; CYIE: 1.29 ± 0.13 mg/mL) [40], and conger eel (*Conger myriaster*) (LGLNGDDVN: 0.687 mg/mL) [42]. HO· could initiate the oxidative stress to damage biomacromolecules in organisms. The finding indicated that RCP2, RCP3, and RCP5 could reduce the HO· damage in biological systems through acting as a radical scavenger.

##### $O_{2}^{-}$·Scavenging Activity

Figure 5C indicates that six CPs (RCP1–RCP6) could strongly scavenge $O_{2}^{-}$· when their concentrations are between 0 and 5.0 mg/mL. The EC_50_ values of RCP1–RCP6 were 7.98 ± 0.34, 0.62 ± 0.07, 0.47 ± 0.05, 0.99 ± 0.10, 0.74 ± 0.06, and 2.43 ± 0.13 mg/mL, respectively. RCP2 and RCP3 showed the highest $O_{2}^{-}$· scavenging ability among six CPs (RCP1–RCP6). In addition, the EC_50_ values of RCP2 and RCP3 were lower than those of antioxidant peptides from protein hydrolysates of blood cockle (*T. granosa*) (EPLSD: 2.04 ± 0.23 mg/mL; WLDPDG: 2.49 ± 0.17 mg/mL; EPVV: 1.69 ± 0.14 mg/mL; CYIE: 2.31 ± 0.15 mg/mL) [40], bluefin leatherjacket (*Navodon septentrionalis*) heads (GVPLT: 2.8819 mg/mL) [36], skipjack tuna (*K. pelamis*) scales (His-Gly-Pro-Hyp-Gly-Glu: 1.19 mg/mL; DGPKGH: 0.71 mg/mL; MLGPFGPS: 1.59 mg/mL) [15], and hairtail (*T. japonicas*) muscle (AKG: 2.538 mg/mL; IYG: 1.355 mg/mL) [37]. $O_{2}^{-}$· is a main cause of oxidative stress because it can generate the highly reactive HO· and create toxic peroxy radicals. Therefore, RCP2 and RCP3 can play important roles in eliminating $O_{2}^{-}$· damage together with SOD in organisms.

#### 2.4.2. Lipid Peroxidation Inhibition Assay

**Figure 6:**
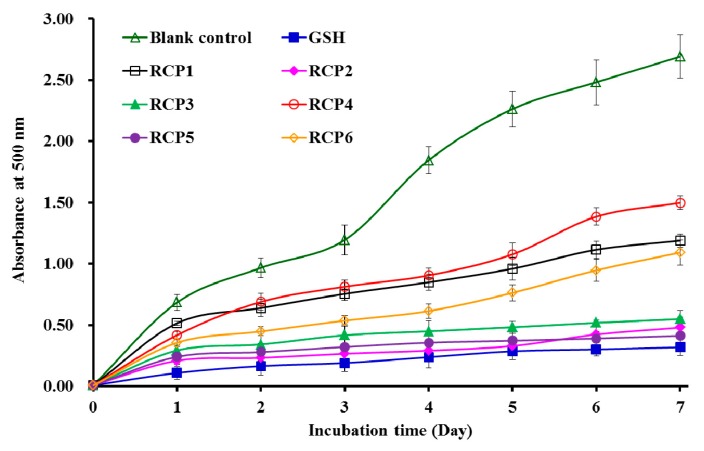
Lipid peroxidation inhibition assays of six collagen peptides (CPs) (RCP1–RCP6) from collagen hydrolysate of redlip croaker (*P. polyactis*) scales. All data are presented as the mean ± SD (*n* = 3).

Figure 6 showed that the absorbance values of RCP2, RCP3, and RCP5 solutions at 500 nm were a little higher than that of the positive control of GSH and significantly lower than those of the blank control (without antioxidant) and the other three CPs (RCP1, RCP4, and RCP6). The data indicated that the abilities of RCP2 and RCP5 on lipid peroxidation inhibition were similar to that of GSH in the linoleic acid model system during 7 days incubation. Lipid oxidation involves in multifarious reactions of lipid radicals and produces various primary and secondary by-products, which significantly affect with food quality, nutritional, and health implications [18,36]. The present results indicated that RCP2 and RCP5 could serve as antioxidants used in food and healthy products because of their significant inhibitory effect on the lipid peroxidation.

#### 2.4.3. Protective Effect of RCP2, RCP3, and RCP5 on Plasmid DNA Damaged by H_2_O_2_

**Figure 7:**
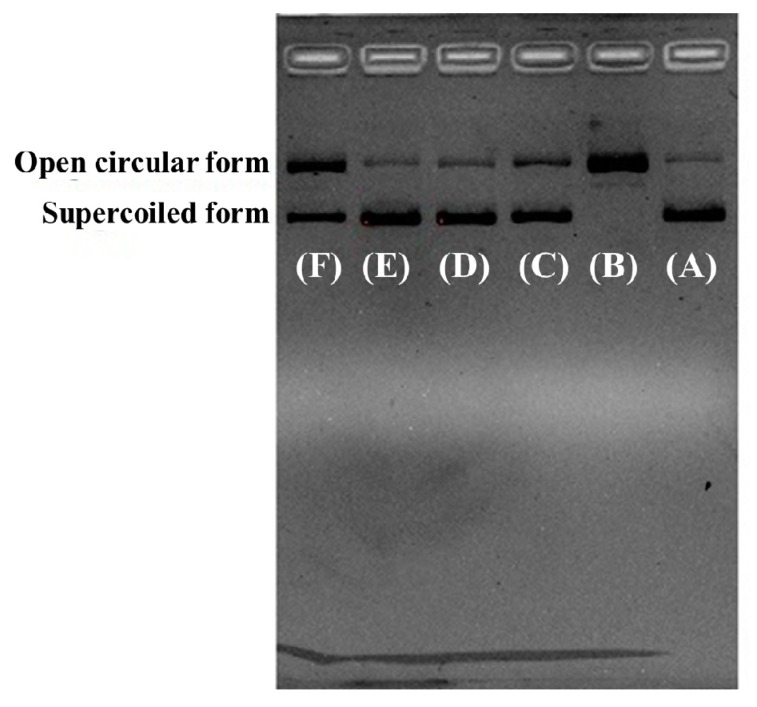
Protective effects of RCP2, RCP3, and RCP5 from collagen hydrolysate of redlip croaker (*P. polyactis*) scales on plasmid DNA damaged by H_2_O_2_ at the concentration of 3.0 mg/mL. (**A**) native pBR322DNA; (**B**) pBR322DNA treated with FeSO_4_ and H_2_O_2_; (**C**) pBR322DNA treated with FeSO_4_, H_2_O_2_ and GSH (1.0 mg/mL); (**D**) pBR322DNA treated with FeSO_4_, H_2_O_2_ and RCP2; (**E**) pBR322DNA treated with FeSO_4_, H_2_O_2_ and RCP3; (**F**) pBR322DNA treated with FeSO_4_, H_2_O_2_, and RCP5. The image is chosen from one of three experiments.

Figure 7A indicated that the plasmid DNA (pBR322DNA) was almost a supercoiled form (SF) under normal conditions (Figure 7A) and turned into an open circular form (OCF) when one phosphodiester chain was cut by HO· (Figure 7B). Figure 7C–F indicated the contents of OCF of plasmid DNA were significantly less than that of Figure 7B, which indicated that RCP2, RCP3, RCP5, and GSH had different protective function on plasmid DNA against HO· damage, and the protective abilities of RCP2 and RCP3 were stronger than that of RCP5. In addition, the images of RCP2 and RCP3 were similar to that of the normal control (Figure 7). Oxidative DNA damage can cause toxicity and mutations, which further lead to cancer and premature aging [43,44]. Therefore, the present results indicated that RCP2 and RCP3 should have strong ability of protecting DNA from the oxidative damage through scavenging HO· generated from the reaction of Fe^2+^ with H_2_O_2_ and/or inhibiting the reaction. 

#### 2.4.4. Cytoprotective Activity of RCP2, RCP3, and RCP5 on H_2_O_2_-Damaged HepG2 Cells

##### Effects of RCP2, RCP3, and RCP5 on the Cell Viability of HepG2 Cells

**Figure 8:**
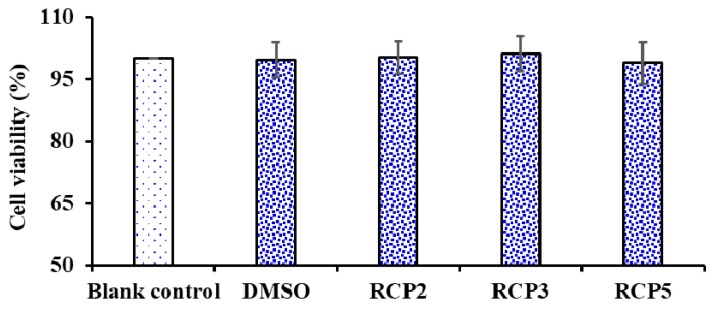
Effects of RCP2, RCP3, and RCP5 from collagen hydrolysate of redlip croaker (*P. polyactis*) scales on the cell viability of HepG2 cells at concentration of 100.0 µM. The data are presented as the mean ± SD (*n* = 3).

As shown in Figure 8, RCP2, RCP3, and RCP5 exhibited no significantly effect on the viability of HepG2 Cells compared to the blank control group at the concentrations of 100.0 µM for 24 h treatment (*p* < 0.05). Cell viability is a determination of living or dead cell rate in a total cell sample, which is generally used for drug screening to detect whether the test molecules have effects on cell proliferation or display direct cytotoxic effects [45]. The result indicated that RCP2, RCP3, and RCP5 showed no significant cytotoxic activity and could serve as the candidate biomolecules for the development of antioxidant food and drugs.

##### Protection of RCP2, RCP3, and RCP5 on H_2_O_2_-Damaged HepG2 Cells

**Figure 9:**
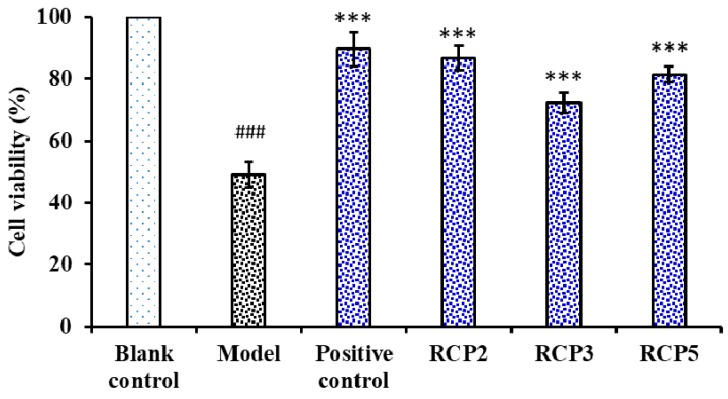
Cytoprotective function of RCP2, RCP3, and RCP5 from collagen hydrolysate of redlip croaker (*P. polyactis*) scales on H_2_O_2_-Damaged HepG2 Cells at concentration of 100.0 µM. Acetylcysteine (NAc) was used as the positive control. The data are presented as the mean ± SD (*n* = 3). ^###^ *p* < 0.001 versus the control group. *** *p* < 0.001 versus the model group (treated with H_2_O_2_ at the concentration of 300 μM).

Figure 9 showed the influences of RCP2, RCP3, and RCP5 on H_2_O_2_-damaged HepG2 cells, and the cell viability of RCP2, RCP3, and RCP5 treated groups were 86.79 ± 3.86%, 72.37 ± 3.35%, and 81.47 ± 2.45% at the concentrations of 100.0 µg/mL, respectively, which were significantly higher than that of model (treated with H_2_O_2_ at the concentration of 300 μM) group (49.58 ± 3.91%) (*p* < 0.001). Oxidative stress reflects an imbalance between the systemic manifestation of ROS and a biological system’s ability to readily detoxify the reactive intermediates or to repair the resulting damage [46]. Long-term oxidative stress can product excess peroxides and ROS, which damage functional molecules within cells and lead to aging, cancer, diabetes, atherosclerosis, inflammatory disorders, chronic fatigue syndrome, and Alzheimer’s and Parkinson’s diseases [47,48]. The finding suggested that RCP2, RCP3, and RCP5 could strongly protect HepG2 cells against H_2_O_2_-induced oxidative damage.

##### Effect of RCP2, RCP3, and RCP5 on the Levels of ROS in H_2_O_2_-Damaged HepG2 Cells

**Figure 10:**
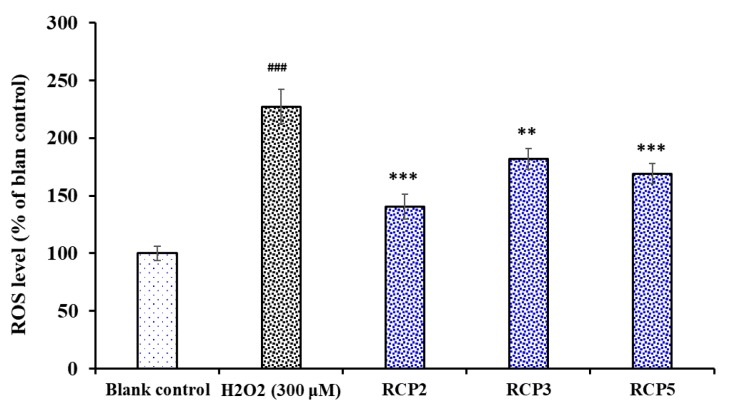
Effects of RCP2, RCP3, and RCP5 on the levels of ROS in H_2_O_2_-damaged HepG2 cells at the concentration of 100 μM. The data are presented as the mean ± SD (*n* = 3). ^###^ *p* < 0.001 versus the blank control group; *** *p* < 0.001 and ** *p* < 0.01 versus the model group (treated with H_2_O_2_ at the concentration of 300 μM).

The effects of RCP2, RCP3, and RCP5 on the levels of ROS in H_2_O_2_-damaged HepG2 cells were determined and presented in Figure 10. The level of ROS observed in the HepG2 exposed to H_2_O_2_ were 227.2 ± 14.8% of the blank control, which was significantly higher than those of the blank control group (*p* < 0.001). As expected, the intracellular ROS levels were significantly decreased by RCP2 (140.6 ± 10.8%), RCP3 (169.1 ± 8.6%), and RCP5 (182.1 ± 8.9%) at the concentrations of 100 μM. In addition, the intracellular ROS levels of RCP2 and RCP3 were significantly lower than that of model group (*P* < 0.001). ROS are generated by different physiological and biochemical oxidative processes in the organisms and associated with numerous physiological and pathophysiological processes [41,42]. High levels of intracellular ROS can oxidize and damage some key biological macromolecules and cytomembrane and play a major role in the pathogenesis of various human diseases [43,44]. The present data indicated that RCP2, RCP3, and RCP5 could protect HepG2 cells against the H_2_O_2_-induced oxidative damage through decreasing the level of ROS.

##### Effects of RCP2, RCP3, and RCP5 on the Level of Intracellular Antioxidant Enzymes and MDA in H_2_O_2_-Damaged HepG2 Cells

**Figure 11:**
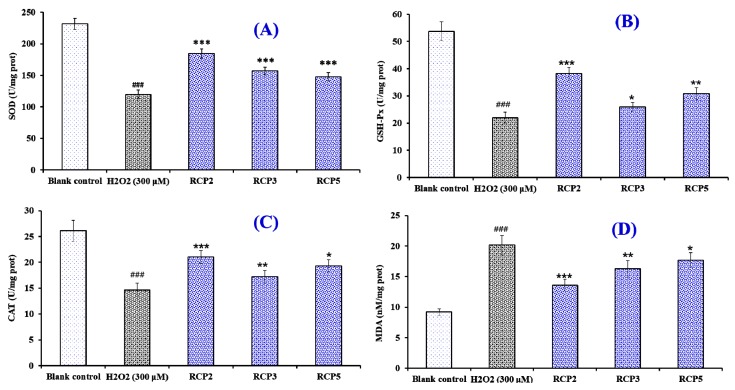
The effects of RCP2, RCP3, and RCP5 on the levels of intracellular antioxidant enzymes (SOD (**A**), CAT (**B**), and GSH-Px (**C**)) and content of malondialdehyde (MDA) (**D**) in H_2_O_2_-damaged HepG2 cells. All data are presented as the mean ± SD (*n* = 3). ^###^ *p* < 0.001 versus the control group; *** *p* < 0.001, ** *p* < 0.01, and * *p* < 0.05 versus the model group (treated with H_2_O_2_ at the concentration of 300 μM).

Figure 11A–C showed the effects of RCP2, RCP3, and RCP5 on the levels of intracellular antioxidant enzymes (SOD, CAT, and GSH-Px). Compared with the blank control group, the levels of SOD, CAT, and GSH-Px were significantly decreased by H_2_O_2_ in the HepG2 cells (*p* < 0.001), which indicated that the oxidative stress induced by H_2_O_2_ seriously damaged the enzymatic antioxidant defense system of HepG2 cells. However, the levels of SOD, CAT, and GSH-Px in HepG2 cells incubated by RCP2, RCP3, and RCP5 significantly higher than that of the H_2_O_2_ damaged group (*p* < 0.05). In addition, the effect of RCP2 on the enzymatic antioxidant defense system was higher than those of RCP3 and RCP5.

Figure 11D showed that the content of MDA (20.14 ± 1.58 nM/mg prot) in the H_2_O_2_-damaged HepG2 cells was significantly (*p* < 0.05) reduced compared with that of the blank control group (9.18 ± 0.54 nM/mg prot). At the concentration of 100 μM, the MDA content of RCP2 incubated group was 13.57 ± 0.96 nM/mg prot, which was lower than those of RCP3 (16.24 ± 1.37 nM/mg prot), and RCP5 (17.65 ± 1.28 nM/mg prot) and the H_2_O_2_ treated groups. Moreover, the MDA content of RCP2, RCP3, and RCP5 were significantly lower than that of the model group (*p* < 0.05). Therefore, RCP2, RCP3, and RCP5 could significantly reduce the oxidative stress injury by restoration of endogenous antioxidation and the decrease of lipid peroxidation.

Cells contain enzymatic and non-enzymatic antioxidant defense systems for combatting and preventing oxidative stress [49]. The enzymatic antioxidant system is mainly comprised of SOD, CAT, and GSH-Px and can hold back the radicals attacking cellular membrane and components [50]. Many dietary antioxidants can help to increase the levels of intracellular antioxidant enzymes to maintain redox homeostasis in cells of the human body [50,51]. MDA is the oxidative metabolite of cell lipid oxidation, and the reduction of oxidative damage in cells was accompanied by the decrease of oxidative metabolites, which give an index to the degree of oxidative damage in cells [52,53]. Recently, antioxidant peptides have received widespread attention due to their nutritional function and activities, especially their ability on increasing the levels and activities of antioxidant enzymes [54]. Tonolo et al. reported that KVLPVPEK derived from milk could activate nuclear factor erythroid 2-related factor (Nrf2) pathway in Caco-2 cells to induce overexpression of phase II enzymes, including thioredoxin 1 (Trx1), thioredoxin reductase (TrxR1), gluthatione reductase (GR), NAD(P)H quinone dehydrogenase 1 (NQO1), and SOD1 [55]. Rahman et al. [56] confirmed that antioxidant peptide of YD1 from the strain *Bacillus amyloliquefaciens* CBSYD1 has strong antioxidant activity, hydrogen atom transfer ability, and the potential to prevent H_2_O_2_-induced oxidative stress in RAW 264.7 cells through the increase of cellular thiol content, GST activity, the expression of antioxidant enzymes and the expression of the redox-sensitive transcription factor Nrf2. Cai et al. [57] found that FPYLRH from the hydrolysate of miiuy croaker (*M. miiuy*) swim bladder could significantly increase the levels of SOD and GSH-Px and decrease the contents of ROS, MDA and nitric oxide (NO) in H_2_O_2_-induced oxidative damage human umbilical vein endothelial cells (HUVECs). In addition, FPYLRH could dose-dependently protect DNA in oxidative damage HUVECs model. The present results indicated that the antioxidant activities of RCP2, RCP3, and RCP5 were similar to KVLPVPEK, YD1, and FPYLRH, which can protect different cells from oxidative stress by enhancing endogenous antioxidant enzyme defense system.

## 3. Experimental Section

### 3.1. Materials

Redlip croaker (*P. polyactis*) scales were kindly supplied by Zhejiang Hailisheng Group Co. Ltd. (Zhejiang, China). Sephadex G-25 and DEAE-52 cellulose were purchased from Shanghai Source Poly Biological Technology Co., Ltd (Shanghai, China). Acetonitrile (ACN) of liquid chromatogram grade and trifluoroacetic acid (TFA) were purchased from Thermo Fisher Scientific Co., Ltd (Shanghai, China). DPPH and GSH were purchased from Sigma–Aldrich (Shanghai, China) Trading Co., Ltd. (Shanghai, China). ASC-RC of the redlip croaker scales were prepared using the method of Wu et al. [32]. DGPEGR (RCP1), GPEGPMGLE (RCP2), EGPFGPEG (RCP3), YGPDGPTG (RCP4), GFIGPTE (RCP5), and IGPLGA (RCP6) with purity higher than 98% were synthesized in China Peptides Co. (Suzhou, China).

### 3.2. Preparation of Protein Hydrolysate from ASC-RC

The dispersions of the ASC-RC (1%, w/v) was separately hydrolyzed by six different proteases according to the designed conditions (Table 4). After that, the hydrolysates were kept in a 95 °C water bath for 15 min and centrifuged at 8000 *g* for 30 min. The resulting supernatants were freeze-dried and stored in a −20 °C refrigerator. The collagen hydrolysate prepared using neutrase was named as RSCH. The DH (%) of hydrolysates was measured using the previous method [30].

### 3.3. Isolation of CPs from RSCH

**Figure 12:**
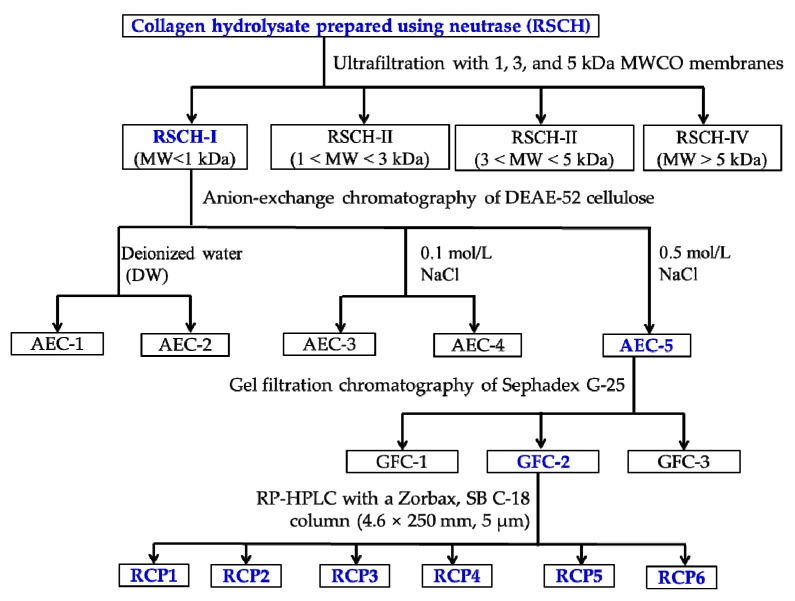
The flow diagram of isolating CPs from the collagen hydrolysate (RSCH) of redlip croaker (*P. polyactis*) scales prepared using neutrase.

CPs were isolated from RSCH using membrane ultrafiltration and serial chromatography (Figure 12). RSCH was fractionated using ultrafiltration (8400, Millipore, Hangzhou, China) with MWCO membranes of 1, 3, and 5 kDa, respectively, and divided into four fractions, including RSCH-I (MW < 1 kDa), RSCH-II (1 < MW < 3 kDa), RSCH-III (3 < MW < 5 kDa) and RSCH-IV (MW > 5 kDa).

RSCH-I solution (5 mL, 40.0 mg/mL) was injected into a DEAE-52 cellulose column (1.6 cm × 80 cm) pre-equilibrated with DW, and stepwise eluted with 150 mL DW, 0.1 M NaCl, 0.5 M NaCl, and 1.0 M NaCl solution at a flow rate of 1.0 mL/min, respectively. Each eluate (5 mL) was monitored at 214 nm. Finally, five fractions (ACE-1 to ACE-5) were pooled and lyophilized on their chromatographic peaks. ACE-5 solution (5 mL, 20.0 mg/mL) was separated on a Sephadex G-25 column (2.6 cm × 160 cm) eluted with DW at a flow rate of 0.6 mL/min. Each eluate (3 mL) was collected and monitored at 214 nm, and the fraction of GFC-2 solution (25 μL, 10.0 mg/mL) was further purified on an Agilent 1260 HPLC system (Agilent Ltd., Santa Rosa, CA, USA) with an Agilent Zorbax, SB C-18 column (4.6 mm × 250 mm). The sample was eluated with a linear gradient of ACN (0%–50% in 0–40 min) in 0.1% TFA at a flow rate of 0.8 mL/min, and six CPs (RCP1 to RCP6) were isolated on the absorbance at 214 nm.

### 3.4. Amino Acid Sequence and Molecular Mass Analysis

The amino acid sequences and molecular masses of six CPs (RCP1 to RCP6) were measured on an Applied Biosystems 494 protein sequencer (Perkin Elmer/Applied Biosystems Inc, Foster City, CA, USA) and a Q-TOF mass spectrometer coupled with an electrospray ionization source, respectively.

### 3.5. Radical Scavenging Assays

The radical scavenging assays of six CPs (RCP1–RCP6) were determined by the previous method [5,30], and the results of the radical scavenging assays were expressed as a half elimination ratio (EC_50_) defined as the concentration where a sample caused a 50% decrease of the initial radical concentration.

#### 3.5.1. DPPH· Scavenging Activity

2.0 mL of samples consisting of distilled water and different concentrations of the analytes were placed in cuvettes, and 500 μL of an ethanolic solution of DPPH (0.02%) and 1.0 mL of ethanol were added. A control sample containing the DPPH solution without the sample was also prepared. In the blank, the DPPH solution was substituted with ethanol. The DPPH· scavenging activity was calculated using the following formula:

DPPH· scavenging activity (%) = (A_c_ + A_b_ − A_s_)/A_c_ × 100%,

where A_s_ is the absorbance rate of the sample, A_c_ is the control group absorbance, and A_b_ is the blank absorbance.

#### 3.5.2. HO· Scavenging Activity

First, 1.0 mL of a 1.865 mM 1,10-phenanthroline solution and 2.0 mL of the sample were added to a screw-capped tube and mixed. Then, 1.0 mL of a FeSO_4_·7H_2_O solution (1.865 mM) was added to the mixture. The reaction was initiated by adding 1.0 mL of H_2_O_2_ (0.03%, v/v). After incubating at 37 °C for 60 min in a water bath, the absorbance of the reaction mixture was measured at 536 nm against a reagent blank. The reaction mixture without any antioxidant was used as the negative control, and a mixture without H_2_O_2_ was used as the blank. The HO· scavenging activity was calculated using the following formula:

HO· scavenging activity (%) = [(A_s_ − A_n_)/(A_b_ − A_n_)] × 100%,

where A_s_, A_n_, and A_b_ are the absorbance values determined at 536 nm of the sample, the negative control, and the blank after the reaction, respectively.

#### 3.5.3. $O_{2}^{-}$· Scavenging Activity

In the experiment, superoxide anions were generated in 1.0 mL of nitrotetrazolium blue chloride (NBT) (2.52 mM), 1.0 mL of NADH (624 mM) and 1 mL of different sample concentrations. The reaction was initiated by adding 1.0 mL of phenazine methosulphate (PMS) solution (120 μM) to the reaction mixture. The absorbance was measured at 560 nm against the corresponding blank after 5-min incubation at 25 °C. The $O_{2}^{-}$· scavenging activity was calculated using the following equation:

$$O_{2}^{-}\cdot \mathrm{scavenging}\;\mathrm{activity}\;(\%)\;=\;\left[\left(A_{c}-A_{s}\right)/A_{c}\right]\times 100\%,$$

where A_c_ is the absorbance without sample and A_s_ is the absorbance with sample.

### 3.6. Lipid Peroxidation Inhibition Assay

The lipid peroxidation inhibition activity of the APs was measured in a linoleic acid model system using the method of Wang et al. [30]. Briefly, a sample (5.0 mg) was dissolved in 10 mL of 50 mM PBS (pH 7.0) and added to 0.13 mL of a solution of linoleic acid and 10 mL of 99.5% ethanol. Then, the total volume was adjusted to 25 mL with deionized water. The mixture was incubated in a conical flask with a screw cap at 40 °C in a dark room, and the degree of oxidation was evaluated by measuring ferric thiocyanate values. The reaction solution (100 μL) incubated in the linoleic acid model system was mixed with 4.7 mL of 75% ethanol, 0.1 mL of 30% ammonium thiocyanate, and 0.1 mL of 20 mM ferrous chloride solution in 3.5% HCl. After 3 min, the thiocyanate value was measured at 500 nm following color development with FeCl_2_ and thiocyanate at different intervals during the incubation period at 40 °C.

### 3.7. Protective Effect on Plasmid DNA

The protective effects of f RCP2, RCP3, and RCP5 on supercoiled plasmid DNA (pBR322) were measured using the previous method [57,58]. In brief, 15 µL of reaction mixtures containing 5 µL of PBS (10 mM, pH 7.4), 2 µL of FeSO_4_ (1.0 mM), 1µL of pBR322 (0.5 µg), 5 µL of the peptide (RCP, RCP3, or RCP5, respectively), and 2 µL of H_2_O_2_ (1.0 mM) were incubated at 37 °C. After 0.5 h incubation, the reaction was terminated by adding 2 µL of loading buffer containing glycerol (50%, v/v), ethylenediaminetetraacetic acid (40 mM), and bromophenol blue (0.05%). The resulted reaction mixtures were subsequently electrophoresed on 1% agarose gel containing 0.5 µg/mL EtBr for 50 min (60 V), and the DNA in the agarose gel was photographed under ultraviolet light.

### 3.8. Cell Culture and Cell Viability Assay

MTT test was performed on the previous method to measure the effects of samples on the cell viability [59,60]. In brief, the HepG2 cells were cultured in DMEM medium contained 10% FBS supplemented with 2 mM l-Glu, and 1% penicillin-streptomycin solution at 37 °C and 5% CO_2_ atmosphere. After 24 h incubation in a 96-well plate (7 × 10^3^ cells/well), the HepG2 cells were cultured at the presence of designed concentrations of peptide solution for 12 h. After that, the wells were washed with phosphate-buffered saline (PBS) for twice and the MTT with the final concentration of 0.5 mg/mL was added into. After 4 h, the formazan crystals formed by active cells were dissolved in 150 µL of dimethyl sulfoxide (DMSO) and the absorbance at 570 nm of the solution was recorded. The cell viability was calculated by the following equation:

Cell viability = (A_sample_/A_control_) × 100%.

### 3.9. Cytoprotective Activity of RCP2, RCP3 and RCP5 on the Oxidative Damaged HepG2 Cells by H_2_O_2_

The assay was performed on the previous method and the H_2_O_2_ concentration of 300 µM was used to build the oxidative damage model of HepG2 cells [48]. In brief, the isolated CPs were dissolved in the Dulbecco’s Modified Eagle’s medium (DMEM) with the concentration of 100.0 µM. The HepG2 cells were grown (6 × 10^4^ cells/well) in a 96-well plate for 24 h. Then the supernatant was aspirated and 100 µL of peptide samples were added into the protection groups respectively for incubating 8 h. After removing peptide samples, H_2_O_2_ was respectively added into the damage and protection groups with the optimal concentration (300 µM) and sequentially incubated for 24 h. Finally, the cell viability was measured and calculated on the method of Section 3.8.

### 3.10. Determination of the Levels of ROS in H_2_O_2_-Induced HepG2 cells

Intracellular ROS accumulation in HepG2 cells was monitored according to the previous method described by Zheng et al. [13]. In brief, HepG2 cells were preincubated with samples at the concentrations of 10, 50, or 100 μM for 12.0 h, and then incubated with H_2_O_2_ at the concentration of 300 µM for 2 h. After that, the cells were washed with PBS and incubated with 10 μM DCFH2-DA in fresh culture medium for 0.5 h. Intracellular ROS levels indicated by DCF fluorescence were quantified on a BD FACS Calibur flow cytometer (BD Biosciences, San Diego, CA, USA) using excitation and emission filters of 488 and 530 nm, respectively. The data were expressed as % of control values.

### 3.11. Determination of the Levels of Antioxidant Enzymes and MDA in H_2_O_2_-Induced HepG2 Cells

The assay was performed according to the previous methods [27,61]. The HepG2 cells were cultured in 6-well plates (1 × 10^6^ cells/well). The isolated peptides (final concentration of 10.0, 50.0, and 100.0 µM, respectively) were added into the protection groups. After that, the damage and protection groups were induced by H_2_O_2_ at the concentration of 300 µM. Finally, 500 mL of cell lysis buffer was added into each well on ice lysed for 0.5 h and centrifuged at 12,000 g, 4 °C for 10 min. The resulted liquid supernatant was followed with cold standby at 4 °C (the indicators should be measured in 6 h). The levels of superoxide dismutase (SOD), CAT, GSH-Px and MDA were measured using assay kits according to the protocols of manufacturer. The results were expressed as units of enzymatic activity per milligram of protein (U/mg prot).

### 3.12. Statistical Analysis

The data are reported as the mean ± standard deviation (SD, *n* = 3). A one-way analysis of variance (ANOVA) test for differences between the means of each group was applied to analyzed data using SPSS 19.0 (Statistical Program for Social Sciences, SPSS Corporation, Chicago, IL, USA).

## 4. Conclusions

In the experiment, scale collagen of redlip croaker (*P. polyactis*) was separately hydrolyzed using six different proteases and six CPs were purified from the collagen hydrolysate prepared using neutrase and identified as DGPEGR, GPEGPMGLE, EGPFGPEG, YGPDGPTG, GFIGPTE, and IGPLGA, respectively. Among them, GPEGPMGLE, EGPFGPEG, and GFIGPTE exhibited the strong radical scavenging activities, lipid peroxidation inhibiting ability, and oxidation-damaged DNA protective activities. Moreover, GPEGPMGLE, EGPFGPEG, and GFIGPTE could protect H_2_O_2_-damaged HepG2 cells from oxidative stress by decreasing ROS and MDA levels and enhancing endogenous antioxidant enzyme (SOD, CAT, and GSH-Px) defense system. These results suggested that the collagen hydrolysate and CPs from redlip croaker scales could serve as functional ingredients in nutraceuticals and pharmaceuticals.

## Figures and Tables

**Table 1:** Degree of hydrolysis (DH, %) and 2,2-diphenyl-1-picrylhydrazyl radical (DPPH·) scavenging activity (%) of collagen hydrolysate of redlip croaker (*P. polyactis*) scale using six kinds of enzymes.

Protease	DH (%)	DPPH Scavenging Activity (10.0 mg/mL, %)
Pepsin	14.93 ± 0.85 ^a^	20.56 ± 0.66 ^a^
Neutrase	21.36 ± 1.18 ^b^	30.97 ± 1.56 ^b^
Papain	16.95 ± 0.76 ^c^	26.33 ± 1.37 ^c^
Trypsin	16.87 ± 1.36 ^c^	24.35 ± 1.71 ^d^
Flavoenzyme	15.67 ± 1.12 ^c^	22.97 ± 1.36 ^d^
Alcalase	18.73 ± 1.25 ^d^	28.06 ± 1.58 ^e^

All data are presented as the mean ± standard deviation (SD, *n* = 3). ^a–e^ Values with the same letters in each column indicate no significant difference (*p* > 0.05).

**Table 2:** Retention time (RT), amino acid sequences, and molecular mass of six CPs (RCP1–RCP6) from collagen hydrolysate of redlip croaker (*P. polyactis*) scale.

No.	RT (min)	Amino Acid Sequence	Theoretical Mass/Observed Mass (Da)
RCP1	15.03	DGPEGR	629.62/629.61
RCP2	17.73	GPEGPMGLE	885.98/885.95
RCP3	19.76	EGPFGPEG	788.80/788.96
RCP4	23.69	YGPDGPTG	762.76/762.75
RCP5	26.85	GFIGPTE	733.81/733.80
RCP6	29.81	IGPLGA	526.63/526.61

**Table 3:** Radical scavenging activity of six CPs (RCP1–RCP6) from collagen hydrolysate of redlip croaker (*P. polyactis*) scales.

No.	EC_50_/(mg/mL)		
	DPPH·	HO·	$O_{2}^{-}$·
RCP1	4.24 ± 0.18 ^a^	1.65 ± 0.11 ^a^	7.98 ± 0.34 ^a^
RCP2	0.59 ± 0.06 ^b^	0.45 ± 0.05 ^b^	0.62 ± 0.07 ^b^
RCP3	0.37 ± 0.05 ^b^	0.33 ± 0.04 ^b^	0.47 ± 0.05 ^b^
RCP4	1.76 ± 0.12 ^c^	1.95 ± 0.14 ^c^	0.99 ± 0.10 ^c^
RCP5	0.45 ± 0.06 ^b^	0.42 ± 0.05 ^b^	0.74 ± 0.06 ^b,c^
RCP6	2.96 ± 0.17 ^d^	4.47 ± 0.24 ^d^	2.43 ± 0.13 ^d^

All data are presented as the mean ± SD (*N* = 3). ^a–d^ Values with the same letters in each column indicate no significant difference of different samples at the same radicals (*p* > 0.05).

**Table 4:** Designed conditions of six kinds of proteases used for hydrolyzing acid-soluble collagen (ASC-RC).

Protease	Temperature (°C)	pH	Total Enzyme Dose (g/100 g Collagen)	Time (h)
Pepsin	37	2.0	2.0	4
Neutrase	60	7.0	2.0	4
Papain	50	6.0	2.0	4
Trypsin	40	8.0	2.0	4
Flavoenzyme	55	7.0	2.0	4
Alcalase	8.0	9.0	2.0	4
